# Large-scale computational drug repositioning to find treatments for rare diseases

**DOI:** 10.1038/s41540-018-0050-7

**Published:** 2018-03-13

**Authors:** Rajiv Gandhi Govindaraj, Misagh Naderi, Manali Singha, Jeffrey Lemoine, Michal Brylinski

**Affiliations:** 1 Department of Biological Sciences, Louisiana State University, Baton Rouge, LA 70803 USA; 2 Division of Computer Science and Engineering, Louisiana State University, Baton Rouge, LA 70803 USA; 3 Center for Computation and Technology, Louisiana State University, Baton Rouge, LA 70803 USA

## Abstract

Rare, or orphan, diseases are conditions afflicting a small subset of people in a population. Although these disorders collectively pose significant health care problems, drug companies require government incentives to develop drugs for rare diseases due to extremely limited individual markets. Computer-aided drug repositioning, i.e., finding new indications for existing drugs, is a cheaper and faster alternative to traditional drug discovery offering a promising venue for orphan drug research. Structure-based matching of drug-binding pockets is among the most promising computational techniques to inform drug repositioning. In order to find new targets for known drugs ultimately leading to drug repositioning, we recently developed *e*MatchSite, a new computer program to compare drug-binding sites. In this study, *e*MatchSite is combined with virtual screening to systematically explore opportunities to reposition known drugs to proteins associated with rare diseases. The effectiveness of this integrated approach is demonstrated for a kinase inhibitor, which is a confirmed candidate for repositioning to synapsin Ia. The resulting dataset comprises 31,142 putative drug-target complexes linked to 980 orphan diseases. The modeling accuracy is evaluated against the structural data recently released for tyrosine-protein kinase HCK. To illustrate how potential therapeutics for rare diseases can be identified, we discuss a possibility to repurpose a steroidal aromatase inhibitor to treat Niemann-Pick disease type C. Overall, the exhaustive exploration of the drug repositioning space exposes new opportunities to combat orphan diseases with existing drugs. DrugBank/Orphanet repositioning data are freely available to research community at https://osf.io/qdjup/.

## Introduction

Repositioning drugs to treat conditions for which they were not originally intended is an emerging strategy offering a faster and cheaper route to develop new treatments compared to traditional drug discovery.^1^ Since repurposed molecules not only have optimized pharmacokinetics, pharmacodynamics, and toxicity profiles, but are also already approved by the U.S. Food and Drug Administration (FDA), this approach speeds up the evaluation of drug candidates in clinical trials at the reduced risk of failure. Drug repositioning is expected to play a major role in the development of treatments for rare, or orphan, diseases defined as those disorders afflicting <200,000 patients in the United States. Even though rare diseases collectively affect more than 350 million people worldwide (https://globalgenes.org/rare-diseases-facts-statistics/), developing new therapeutics for their small individual markets is not profitable enough to warrant commercial interest.^2^ On that account, many countries passed orphan drug legislation, such as the Orphan Drug Act of 1983 in the U.S., in order to provide financial inducements in terms of the market exclusivity and reduced development costs. Legislators work on the Orphan Product Extensions Now Accelerating Cures and Treatments (OPEN) Act to extend the market exclusivity for repurposing already approved drugs to treat rare diseases,^3^ signifying the importance of drug repositioning to orphan disease research.

It is noteworthy that most repurposed drugs are the result of serendipitous observations made either in the lab or during clinical tests. Sildenafil is perhaps the most recognized example of a repositioned compound. Originally developed to treat hypertension and angina pectoris in the 1980s, it was later repurposed to erectile dysfunction and pulmonary arterial hypertension.^4^ Other examples are amantadine and memantine. The former was introduced in the 1960s as a prophylactic agent in respiratory infections.^5^ A few years later, a patient with Parkinson’s disease experienced a dramatic improvement in her symptoms during the daily administration of amantadine for influenza prophylaxis.^6^ This anecdotal observation stimulated research on using amantadine and other members of the aminoadamantane class of molecules to treat neurological diseases. Indeed, amantadine is presently approved by the FDA as both an antiviral and an antiparkinsonian drug. Structurally similar to amantadine, memantine was also synthesized in the 1960s as a putative hypoglycemic agent, though it was found to be devoid of such activity. It was later discovered that memantine is an uncompetitive antagonist of glutamatergic N-methyl-D-aspartate (NMDA) receptors^7^ and currently, memantine is used to treat moderate to severe Alzheimer-type dementia.^8^

A clear necessity for rational approaches to find alternative indications for existing therapeutics has stimulated the development of computational methods for drug repositioning.^9^ Many currently available algorithms exploit the fact that proteins with similar pockets tend to have similar functions and recognize similar molecules.^10^ For instance, the sequence-order independent profile-profile alignment (SOIPPA) program employs Delaunay tessellation of Cα atoms and geometric potentials to compare binding pockets.^11^ Further, SiteAlign measures distances between druggable pockets with cavity fingerprints constructed by projecting eight topological and physicochemical properties onto a multidimensional, discretized space.^12^ Both SOIPPA and SiteAlign have been used in drug repurposing, for example, SOIPPA helped reveal new targets for entacapone and tolcapone,^13^ whereas SiteAlign detected the cross-reaction of protein kinase inhibitors with a protein regulating neurotransmitter release in the synapse.^14^

Notwithstanding the success of existing methods to recognize similar pockets, many of these algorithms perform well only against the experimental structures of proteins complexed with small molecules. Utilizing datasets of target structures with predicted binding sites poses a formidable challenge for pocket matching programs because of inevitable inaccuracies in the annotation of binding residues. To alleviate this issue, we recently developed *e*MatchSite, which offers a high tolerance to residue misannotations and, to some extent, structure imperfections in ligand-binding regions.^15,16^ In this communication, we combine *e*MatchSite and structure-based virtual screening (VS) with AutoDock Vina^17^ in order to enhance the accuracy of binding site matching. Subsequently, we demonstrate the effectiveness of *e*MatchSite/VS for a kinase inhibitor, which is a confirmed candidate for repositioning to synapsin Ia. Next, this methodology is employed to explore new opportunities to combat orphan conditions through a large-scale repositioning of existing drugs to proteins linked to rare diseases.^18^ The results are discussed with respect to the structural data recently released in the Protein Data Bank (PDB)^19^ for tyrosine-protein kinase HCK, as well as a possibility to repurpose a steroidal aromatase inhibitor to treat Niemann-Pick disease type C. Overall, the protocol combining protein structure modeling, binding site prediction and matching, and structure-based virtual screening holds a significant promise to systematically explore the drug repositioning space at the systems level.

## Results and discussion

### Integrating binding site matching with virtual screening

Although the primary application of VS is to identify potentially bioactive molecules, it can also be used to indirectly measure the similarity between binding sites.^20^ Specifically, VS is conducted against a pair of target pockets and then a statistical dependence between the ranking of library compounds is evaluated by Spearman’s *ρ* rank correlation coefficient. A high positive Spearman’s ρ indicates that two binding sites are chemically similar, i.e., tend to bind similar compounds (Supplementary Text S1). Here, we employ structure-based VS with Vina to increase the accuracy of *e*MatchSite detecting similar binding sites. Since many proteins associated with rare diseases are yet to be experimentally annotated, repositioning drugs to orphan proteins is generally dependent on the accuracy of pocket matching conducted against computationally predicted binding sites. On that account, we run both *e*MatchSite and Vina on pockets predicted by *e*FindSite for ligand-bound and unbound structures in the Huang dataset.^21^

**Fig. 1:**
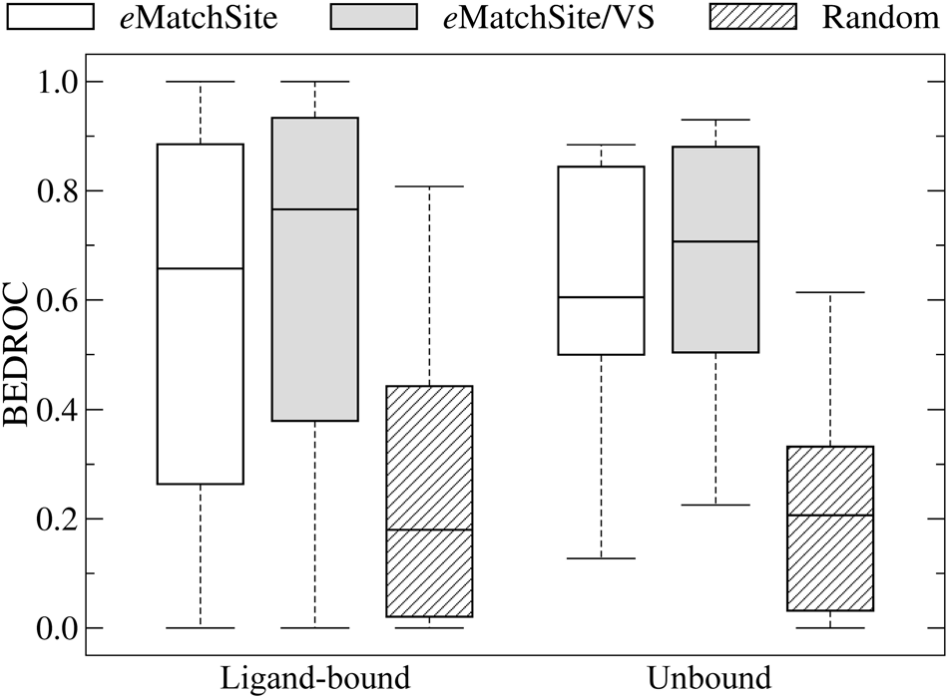
Performance assessment for the recognition of pockets binding similar ligands. The performance of *e*MatchSite alone and including virtual screening, labeled as *e*MatchSite/VS, is evaluated with BEDROC against the Huang dataset and compared to that of a random classifier. The Huang dataset contains ligand-bound and unbound structures. Boxes end at the quartiles Q_1_ and Q_3_, the horizontal line in a box is the median, and whiskers point at the farthest points that are within 3/2 times the interquartile range

The performance is assessed in Fig. 1 with the Boltzmann-Enhanced Discrimination of Receiver Operating Characteristic (BEDROC, Supplementary Text S2) devised to statistically evaluate the early recognition capabilities of binary classifiers.^22^ Using the bound structures, the median BEDROC score for *e*MatchSite alone is 0.66, which increases to 0.77 when it is combined with VS. As expected, the performance for unbound structures is somewhat lower compared to bound structures, however, including VS brings about similar improvements. The median BEDROC for unbound structures increases from 0.60 for *e*MatchSite to 0.71 for *e*MatchSite/VS. For comparison, the performance of a random classifier is notably lower with the median BEDROC values of 0.18 for bound and 0.21 for unbound structures. Overall, combining pocket matching with methodologically orthogonal structure-based VS is an effective strategy to increase the accuracy of detecting pockets binding similar ligands regardless of the conformational state of target proteins.

### Example of a validated candidate for repositioning

**Fig. 2:**
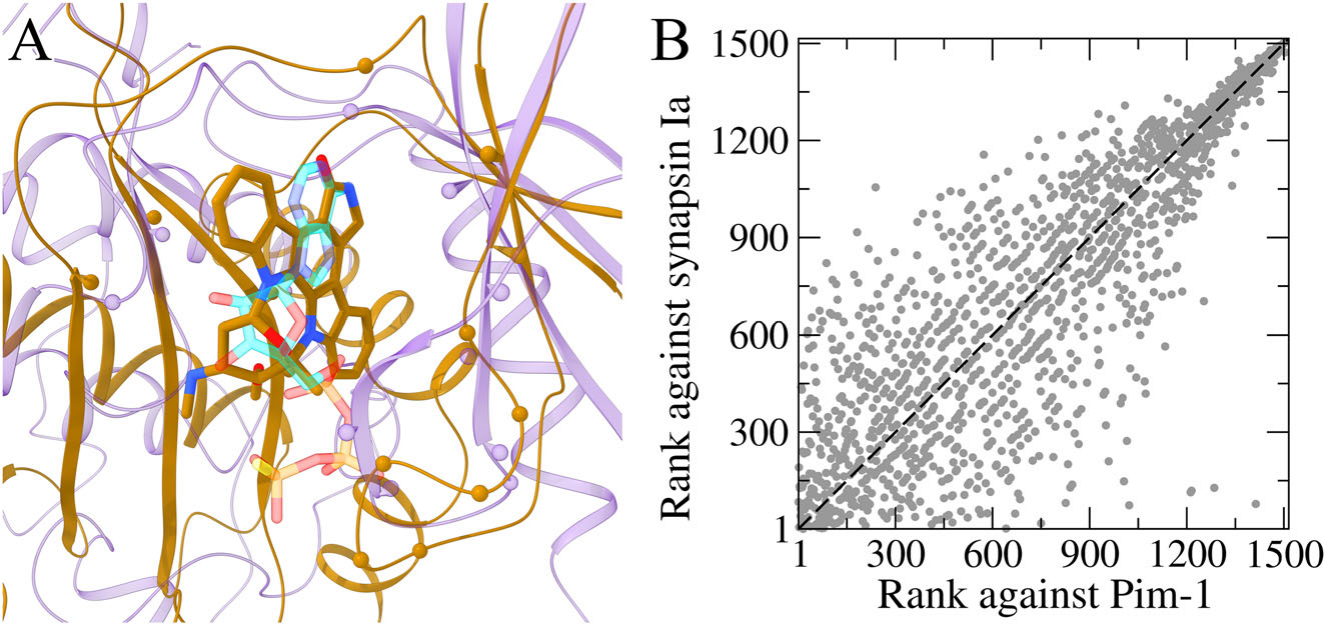
Example of a successful drug repositioning with *e*MatchSite. Staurosporine is repositioned from serine/threonine-protein kinase Pim-1 to synapsin Ia. **a** The local superposition of binding pockets according to the *e*MatchSite alignment. The computer-generated model of the primary target, Pim-1, is represented by transparent purple ribbons, whereas the model of the off-target, synapsin Ia, is represented by solid gold ribbons. Binding residues predicted by *e*FindSite are shown as spheres. The repositioned drug is presented as solid sticks colored by atom type (gold–carbon, red–oxygen, blue–nitrogen). ATP bound to synapsin Ia is shown as transparent sticks colored by atom type (teal–carbon, red–oxygen, blue–nitrogen, yellow–sulfur, peach–phosphorus). **b** A scatter plot for the correlation of ranks from virtual screening conducted by Vina. Each dot represents one library compound, whose ranks against the primary target and off-target are displayed on *x* and *y* axes, respectively. A dashed black line is the diagonal corresponding to a perfect correlation

The capability of *e*MatchSite to correctly recognize similar ligand-binding sites in primary and off-targets is demonstrated for a confirmed candidate for repositioning. Here, we conduct pocket matching followed by VS against weakly homologous protein models constructed by *e*Thread with binding sites predicted by *e*FindSite. This procedure is essentially the same as that employed to reposition existing drugs to proteins linked to rare diseases.^18^ The example shown in Fig. 2 is the cross-reaction of staurosporine, a pan-kinase inhibitor,^23^ with synapsin Ia, an ATP-binding protein regulating neurotransmitter release in the synapse.^14^ A weakly homologous model of the primary target for staurosporine, the human proto-oncogene serine/threonine-protein kinase Pim-1, was built based on the structure of the murine AMP-activated protein kinase (PDB-ID: 5ufu, chain A, 31.3% sequence identity to Pim-1).^24^ This model has a Template Modeling (TM)-score^25^ of 0.86 and a Cα-root-mean-square deviation (RMSD)^26^ of 5.47 Å against the crystal structure of Pim-1 (PDB-ID: 1yhs, chain A),^27^ with a Matthews correlation coefficient (MCC)^28^ between predicted and staurosporine-binding residues of 0.67. The TM-score and Cα-RMSD are described in the Supplementary Text S3. The model of synapsin Ia constructed based on a remote template α-aminoadipate-LysW ligase LysX (PDB-ID: 3vpd, chain A, 22.8% sequence identity to synapsin Ia)^29^ has a TM-score of 0.67 and a Cα-RMSD of 9.03 Å against the crystal structure of synapsin Ia (PDB-ID: 1aux, chain A).^30^

Although Pim-1 and synapsin Ia have globally different sequences (a sequence identity of 15.5%) and structures (a TM-score of 0.32), *e*MatchSite predicted that pockets in both models are in fact highly similar with an *e*MS-score (Supplementary Text S4) of 0.97. Figure 2a presents the local superposition of binding sites in Pim-1 (purple ribbons and spheres) and synapsin Ia (gold ribbons and spheres) models resulting in a Cα-RMSD of 2.47 Å over 13 aligned residues. In addition to staurosporine repositioned to synapsin Ia (solid gold sticks), Fig. 2a includes an ATP-γS molecule bound to the active site of synapsin Ia (transparent teal sticks). Encouragingly, staurosporine transferred according to the local Pim-1→synapsin Ia alignment adopts an orientation closely resembling those of typical ATP-competitive inhibitors bound to protein kinases.^31^ Further, Spearman’s ρ calculated for ranks assigned by Vina is as high as 0.86 (Fig. 2b) strongly indicating that these pockets are in fact chemically similar. Indeed, competition experiments of staurosporine against ATP-γS confirmed its nanomolar binding to synapsin Ia^14^ corroborating the model constructed by *e*MatchSite.

### Repositioning of DrugBank compounds to Orphanet proteins

**Fig. 3:**
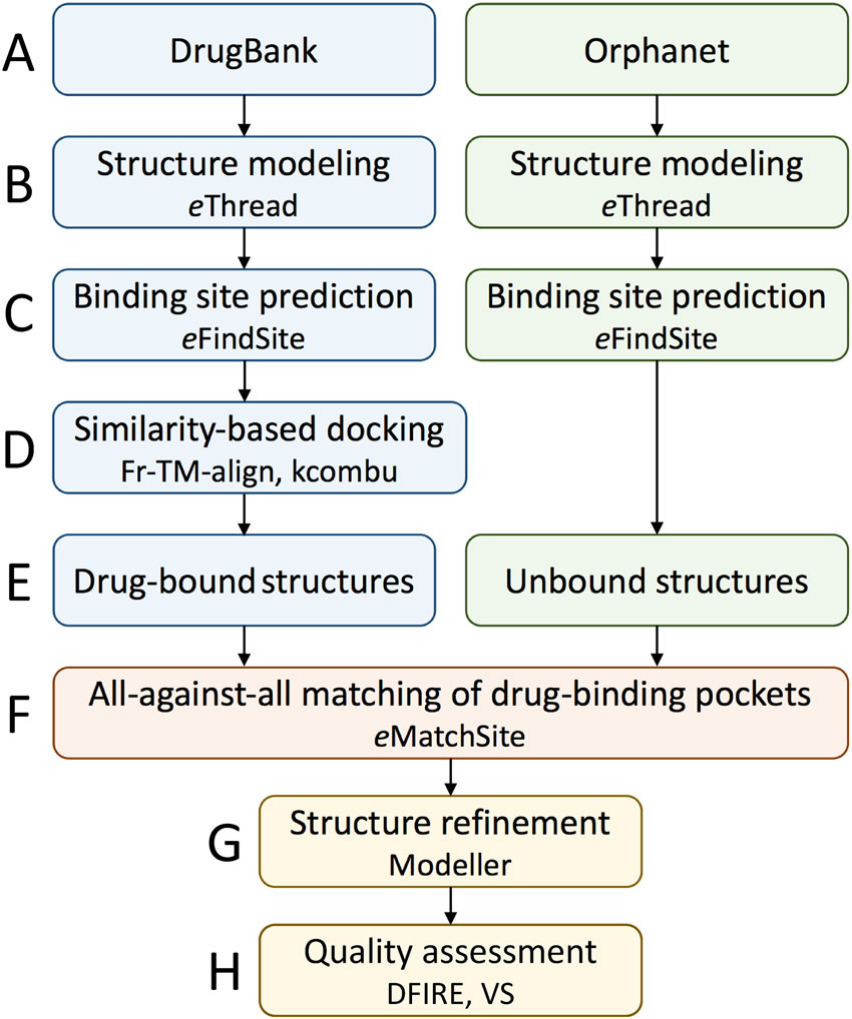
Flowchart of the drug repositioning procedure devised in this study. For protein sequences from DrugBank and Orphanet (**a**), template-based structure modeling is conducted with *e*Thread to construct 3D models (**b**). Protein models are subsequently annotated by *e*FindSite with drug-binding sites (**c**). A similarity-based ligand docking is performed for DrugBank drug-protein pairs, i.e., a globally similar template is aligned onto the target structure with Fr-TM-align and then the drug is aligned onto the template-bound ligand with KCOMBU (**d**). The modeling procedure produces drug-bound structures for DrugBank and unbound structures for Orphanet proteins (**e**). Next, all-against-all matching of drug-binding pockets in DrugBank and Orphanet proteins is conducted with *e*MatchSite (**f**). The DrugBank compound is transferred to the Orphanet model when the similarity of binding pockets is sufficiently high and the resulting complex is refined (**g**). Finally, the quality of final Orphanet complex models is assessed with DFIRE and virtual screening (**h**)

The repositioning procedure was developed in our previous study^18^ and it is summarized in Fig. 3. Full-chain structures of proteins from DrugBank^32^ and Orphanet (Fig. 3a) are modeled with *e*Thread^33^ (Fig. 3b) followed by the annotation of ligand-binding sites with *e*FindSite^34^ (Fig. 3c). Next, drug-target complexes are constructed for DrugBank proteins with a two-step similarity-based docking procedure employing Fr-TM-align^35^ and KCOMBU^36^ (Fig. 3d). This protocol generates drug-bound structures for DrugBank and unbound structures for Orphanet proteins (Fig. 3e). Subsequently, all DrugBank pockets are compared against all Orphanet pockets with *e*MatchSite^15,16^ and drugs are transferred from DrugBank to Orphanet proteins for significant matches (Fig. 3f). Finally, Orphanet drug-target complexes are refined with Modeller^37^ (Fig. 3g) and subjected to quality assessment with Distance-scaled Finite Ideal-gas REference (DFIRE)^38^ and VS^17^ (Fig. 3h).

All-against-all pockets matching conducted with *e*MatchSite for DrugBank and Orphanet proteins produced 320,856 binding site alignments, 5.6% of which yield a statistically significant *e*MS-score. It is noteworthy that the average TM-score between matched DrugBank and Orphanet targets is as low as 0.27 ± 0.10 indicating that in the majority of cases, existing drugs are repositioned from proteins having globally unrelated structures. Based on 18,145 confident local alignments reported by *e*MatchSite, 31,142 unique putative complexes between DrugBank compounds and Orphanet proteins have been modeled. An analysis of the DrugBank→Orphanet repositioning data reveals that 381 existing drugs could be repurposed to target as many as 761 Orphanet proteins. These proteins link to 980 orphan diseases representing 32 classes including (ten the most common classes) 923 genetic, 428 neurological, 377 inborn errors of metabolism, 266 developmental anomalies during embryogenesis, 170 eye, 117 skin, 102 bone, 93 neoplastic, 92 endocrine, and 85 hematological disorders.

#### Repositioning multiple drugs through a single alignment

**Fig. 4:**
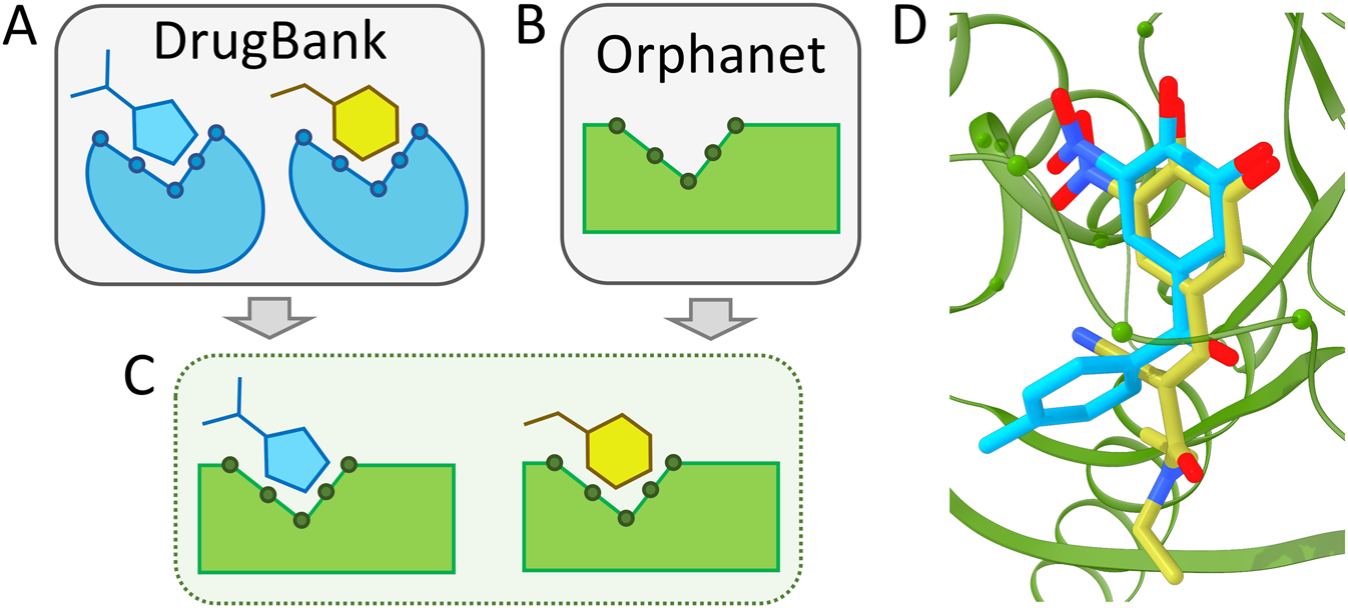
Multiple drugs repositioned through a single pocket alignment. Schematic of (**a**) a single DrugBank target binding two drugs (teal and yellow), (**b**) an Orphanet target (green), and (**c**) two modeled complexes of DrugBank drugs and an Orphanet protein (teal-green and yellow-green). **d** A real example of tolcapone (teal) and entacapone (yellow) repositioned to guanine nucleotide-binding protein subunit alpha-11 (green) based on its local alignment with catechol O-methyltransferase. Non-carbon ligand atoms in panel (**d**) are colored by atom type (blue–nitrogen, red–oxygen)

Drug repositioning conducted in this study includes two kinds of special cases. Figure 4 illustrates the first situation, in which complexes between multiple drugs (Fig. 4a) and an Orphanet target (Fig. 4b) are modeled based on a single pocket alignment. Employing this approach generates a series of structure models of drugs transferred from a DrugBank target to the binding site of an Orphanet protein (Fig. 4c). For instance, catechol O-methyltransferase (COMT) produces a significant local alignment with guanine nucleotide-binding protein subunit alpha-11 (GNA11), associated with a rare disease, autosomal dominant hypocalcemia (ADH) or hypoparathyroidism^39^ (ORPHA:428, GARD:2877). This condition is characterized by low levels of calcium in the blood and an imbalance of other molecules, such as phosphate and magnesium, leading to a variety of symptoms, although about half of affected individuals have no associated health problems.^40^ ADH is primarily caused by mutations of a gene encoding the calcium-sensing receptor, however, activating mutations in GNA11 have also been reported.^41,42^ A binding site predicted in GNA11 by *e*FindSite aligns well to a pocket binding tolcapone and entacapone in COMT with an *e*MS-score of 0.97 and a Cα-RMSD of 4.5 Å calculated over 14 aligned binding residues. Based on this single alignment, tolcapone and entacapone, COMT inhibitors used as adjuncts to levodopa/carbidopa medication in the treatment of Parkinson’s disease,^43,44^ could be repositioned to GNA11. Figure 4d shows the putative binding poses of both compounds in the binding pocket of GNA11 modeled based on the local COMT→GNA11 alignment reported by *e*MatchSite. Interaction energies with GNA11 reported by DFIRE for tolcapone and entacapone are −355.7 and −311.7, respectively. For comparison, the interaction energies with COMT are −283.7 for tolcapone and −310.9 for entacapone. Overall, these results indicate that both molecules may favorably bind to GNA11 producing stable, low-energy assemblies.

#### Construction of multiple models of a single complex

**Fig. 5:**
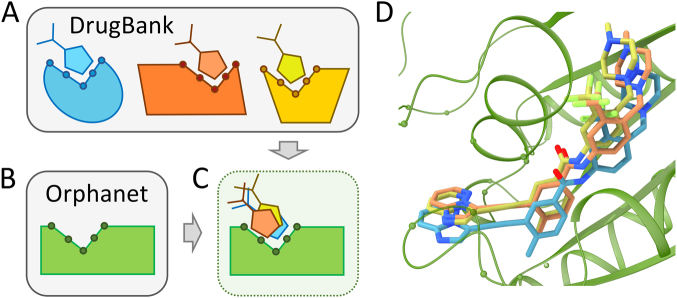
Multiple models of a single drug-target complex constructed based on multiple pocket alignments. Schematic of (**a**) three DrugBank targets binding the same drug (teal, orange, and yellow), (**b**) an Orphanet target (green), and (**c**) three poses of a DrugBank drug within the binding site of an Orphanet protein (teal/orange/yellow-green) modeled from different pocket alignments. (**d**) A real example of ponatinib repositioned to Ras-related protein Rab-23 (green) based on its local alignment with Lck/Yes-related novel protein tyrosine kinase (ponatinib is teal), lymphocyte cell-specific protein-tyrosine kinase (ponatinib is orange), and proto-oncogene tyrosine-protein kinase Src (ponatinib is yellow). Non-carbon ligand atoms in panel (**d**) are colored by atom type (blue–nitrogen, red–oxygen)

The second special case is the modeling of a single complex based on multiple pocket alignments. More than one structure model of a drug repositioned to the Orphanet protein can be constructed if this drug has multiple targets in DrugBank producing significant pocket alignments with the Orphanet protein. This procedure is illustrated in Fig. 5. Figure 5a shows three DrugBank targets binding the same compound, colored in blue, orange and yellow. Assuming that pockets for this drug in all three proteins align to a binding site in an Orphanet target colored in green (Fig. 5b), three independent structure models can be constructed (Fig. 5c). An example is ponatinib, a novel inhibitor of Bcr-Abl tyrosine kinase developed to treat chronic myeloid leukemia and Philadelphia chromosome-positive acute lymphoblastic leukemia.^45^ Ponatinib is a multi-targeted compound, which in addition to its primary target, Abelson tyrosine-protein kinase 1, binds to 14 other macromolecules according to DrugBank.^32^ Binding sites of three of these proteins, Lck/Yes-related novel protein tyrosine kinase (LYN), lymphocyte cell-specific protein-tyrosine kinase (LSK), and proto-oncogene tyrosine-protein kinase Src (SRC), produce significant local alignments with a drug-binding pocket predicted in Ras-related protein Rab-23 (RAB23). The corresponding *e*MS-score/Cα-RMSD values reported by *e*MatchSite for these alignments are 0.97/3.8, 0.98/3.7, and 0.98/3.8 Å, respectively. According to Orphanet, RAB23 is associated with Carpenter syndrome^46,47^ (ORPHA:65759, GARD:6003), a very rare disease with approximately 40 cases described in the literature.^48^ The repositioning of ponatinib to RAB23 can, therefore, be carried out through kinases LSK, LYN, and SRC, resulting in three independent models of a ponatinib-RAB23 complex structure. Figure 5d shows that the binding poses of ponatinib in the RAB23 pocket are very similar across these models. The heavy-atom RMSD between ponatinib molecules is 2.1 Å for LSK- and LYN-based models, 0.7 Å for LSK-based and SRC-based models, and 2.3 Å for LYN-based and SRC-based models, with similar drug-protein interactions present in all models (Supplementary Fig. S1). The interaction energy between ponatinib and RAB23 reported by DFIRE for LSK-, LYN-, and SRC-based models are −829.7, −727.7, and −723.6, respectively. These values are even lower than those calculated for the parent complexes of ponatinib and LSK (−587.9), LYN (−571.9), and SRC (−586.9) suggesting that ponatinib may form favorable interactions with the binding residues of RAB23.

Multiple structure models of the same complex of a drug repositioned to the Orphanet target can be used to estimate the confidence of the large-scale modeling reported in this study. Specifically, employing different DrugBank proteins to transfer the same drug to the Orphanet target should, in principle, produce similar complex models. To test this assumption, we selected 4878 drugs repositioned to Orphan targets by matching binding sites of multiple DrugBank proteins. Supplementary Fig. S2 shows that up to 20 different models can be constructed for some drugs, however, two and three models are generated for the majority of cases (52.4 and 21.9%, respectively). Next, we identified the most typical binding pose of each drug in the pocket of an Orphanet protein by calculating a ligand heavy-atom RMSD against all other models of the same drug-target complex. The distribution of these RMSD values across 4878 DrugBank drugs repositioned to Orphanet targets is shown as inset in Supplementary Fig. S2. Encouragingly, the RMSD for most compounds is relatively low with a median value of 3.6 Å. One should keep in mind that these complex structures are constructed from the computer-generated models of target proteins with computationally predicted ligand-binding sites, and drug molecules are transferred according to fully sequence order-independent pocket alignments.

### Binding affinity prediction for repositioned drugs

**Fig. 6:**
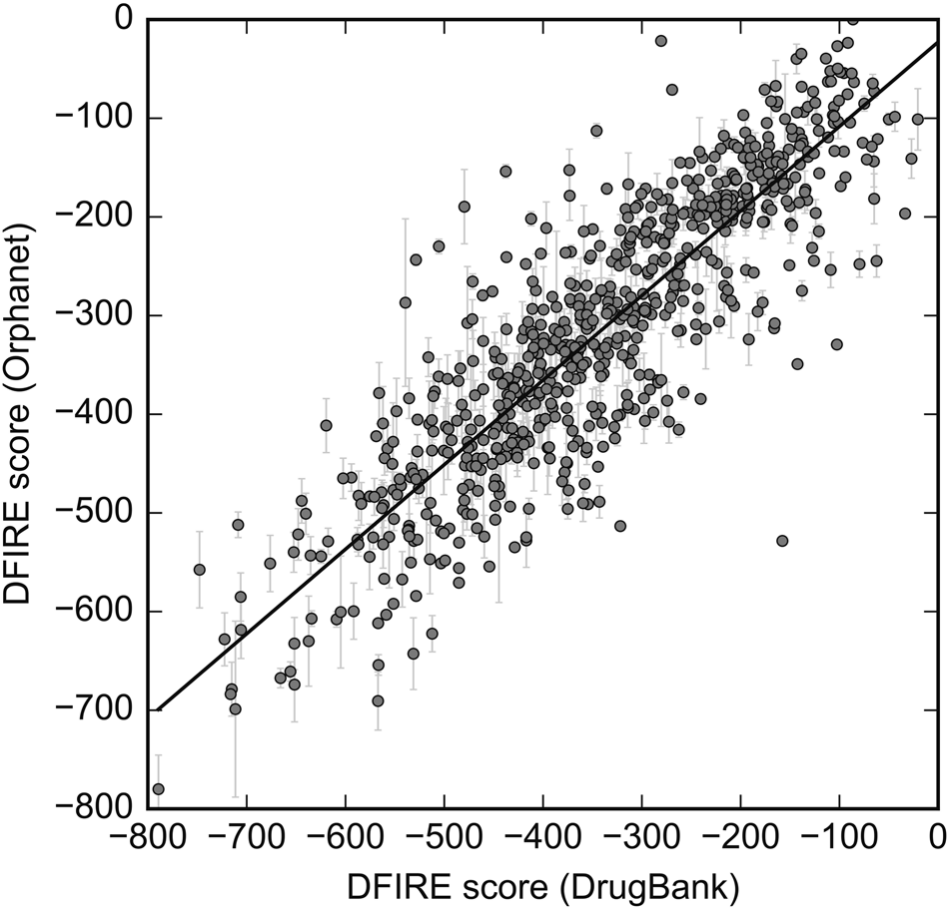
Correlation between interaction energies calculated for DrugBank and Orphanet complex models. Each gray dot represents a drug-target pair from the DrugBank database, whose DFIRE score is displayed on the *x*-axis. Since a drug can be repositioned to multiple Orphanet proteins, the mean DFIRE score ± standard error is displayed on the *y*-axis. Linear regression is shown as a solid line

We also evaluate the binding affinity of drugs repositioned to Orphanet proteins in comparison with their complexes with primary targets from DrugBank. Figure 6 shows the relation between interaction energies estimated by DFIRE for DrugBank and Orphanet complexes. The DFIRE statistical potential is described in the Supplementary Text S5. Because a single drug-target complex from DrugBank can be used to reposition the bound drug molecule to multiple Orphanet proteins, mean scores and the corresponding standard errors of the mean are plotted on the *y*-axis. Encouragingly, DFIRE energies calculated for DrugBank and Orphanet complexes involving the same drug are highly correlated with a Pearson correlation coefficient of 0.86. This analysis indicates that the interaction strength of drug molecules repositioned to Orphanet proteins is generally comparable to that calculated for their complexes with primary targets. Therefore, those pairs of DrugBank and Orphanet proteins producing statistically significant pocket alignments also share similarities with respect to ligand binding as independently evaluated with knowledge-based statistical potentials.

### Validation against a recently determined X-ray structure

**Fig. 7:**
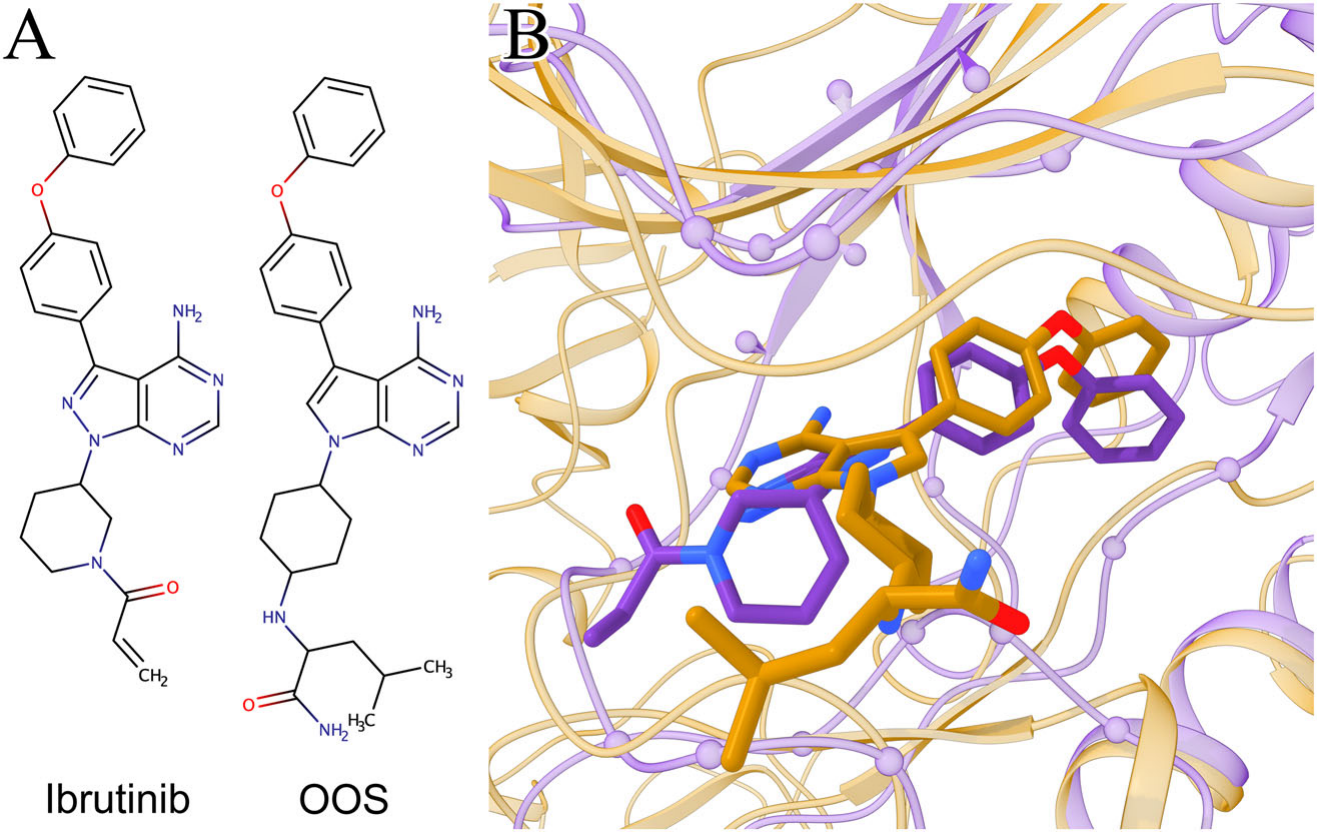
Example of a recently determined structure corroborating repositioning prediction by *e*MatchSite. Ibrutinib repositioned from tyrosine-protein kinase BTK to tyrosine-protein kinase Blk is compared to the X-ray structure of tyrosine-protein kinase HCK complexed with ligand OOS. **a** Chemical structures of the repositioned drug, ibrutinib, and the co-crystallized ligand, OOS. **b** The modeled structure of the ibrutinib-Blk complex, colored in purple, is globally superposed onto the experimental OOS-HCK structure, colored in gold. Proteins are shown as ribbons, ligands as sticks, and binding residues predicted by *e*FindSite in Orphanet models as spheres. Non-carbon atoms in ligands are colored by atom type (red–oxygen, blue–nitrogen, yellow–sulfur)

Repositioning prediction by *e*MatchSite is further validated against a complex structure released in the PDB several months after the modeling was completed. Figure 7 shows ibrutinib (DrugBank-ID: DB09053), an anti-cancer drug primarily targeting B-cell malignancies,^49^ predicted to bind to proto-oncogene, Src family tyrosine kinase Blk (UniProt-ID: P51451). According to Orphanet, Blk is linked to maturity-onset diabetes of the young (MODY, ORPHA:552, GARD:3697)^50^ caused by mutations in at least 13 genes, 5 of which are placed within 100 kb corresponding to the Blk gene.^51^ Nonetheless, a reassessment study showed that Blk mutations, A71T in particular, unlikely cause highly penetrant MODY and may weakly influence type 2 diabetes risk in the context of obesity.^52^ More recently, it was discovered that malignant T cells in the majority of patients with the cutaneous T-cell lymphoma (CTCL) display the ectopic expression of Blk.^53^ Since Blk functions as an oncogene promoting the proliferation of malignant T cells, it is a potential therapeutic target in CTCL.^54^

Although the full-length experimental structure of Blk is unavailable, a confident model of Blk, whose estimated Global Distance Test (GDT)-score^55^ (Supplementary Text S2) is 0.72, was constructed by *e*Thread based on proto-oncogene tyrosine-protein kinase Src (PDB-ID: 1y57, chain A, 64% sequence identity to Blk).^56^ Further, the binding site annotated in the Blk model by *e*FindSite with a 99.2% confidence (Supplementary Text S6) was matched to the ibrutinib-binding pocket in tyrosine-protein kinase BTK with a high *e*MS-score of 0.99. In October 2017, tyrosine-protein kinase HCK co-crystallized with a 7-substituted pyrrolo-pyrimidine inhibitor, OOS (PDB-ID: 5h0e, chain A), sharing 69.7% sequence identity with Blk, was released in the PDB.^57^ Figure 7a shows that ibrutinib and OOS have very similar chemical structures with a Tanimoto coefficient^58^ (TC, Supplementary Text S7) of 0.61 and 27 common atoms.

The global superposition of the modeled ibrutinib-Blk and experimental OOS-HCK structures is presented in Fig. 7b. The Blk model (purple ribbons) has a globally correct structure with a TM-score of 0.86 and a Cα-RMSD of 2.25 Å calculated against HCK (gold ribbons) over the kinase domain. Further, binding residues were accurately predicted by *e*FindSite in the Blk model (purple spheres) with a MCC of 0.60 against OOS-binding residues in the HCK complex structure. Encouragingly, the binding pose of ibrutinib repositioned to Blk based on the local BTK→Blk alignment closely resembles the conformation of OOS in HCK. The RMSD calculated over equivalent non-hydrogen atoms of these compounds is 2.57 Å and 1.43 Å upon the superposition of target proteins and ligands, respectively. Despite the fact that matching binding sites in a sequence-order independent manner is a challenging task, the modeled ibrutinib-Blk complex is noticeably similar to the experimental OOS-HCK structure recently released in the PDB.

### Niemann-Pick disease, type C and exemestane

Niemann-Pick disease, type C (NPC, ORPHA:646) is a fatal hereditary disorder characterized by the accumulation of low-density, lipoprotein-derived cholesterol in lysosomes causing hepatosplenomegaly and severe progressive neurological dysfunction. Mutations in either of two lysosomal proteins, Niemann-Pick disease types C1 (NPC1) or C2 (NPC2), interrupt sterol transport from late endosomes and lysosomes to other cellular organelles resulting in cholesterol accumulation in lysosomes and the fatal NPC disease.^59^ As many as 22 mutations in NPC2 are associated with orphan NPC diseases, including adult, juvenile, late infantile, and severe early infantile neurologic onset. In particular, V30M, V39M, C47F, S67P, C93F, C99R, and P120S mutations in NPC2 have an effect on cholesterol binding.^60–64^ Furthermore, mutations of M79, V81, and V83 block sterol transport making NPC2 a promising drug target to treat NPC diseases.^65^ Interestingly, *e*MatchSite detected a significant structure similarity between the cholesterol-binding pocket of NPC2 and the steroid-binding pocket of cytochrome P450 aromatase (CYP19A1), an enzyme involved in the biosynthesis of aromatic C18 estrogen from C19 androgen. CYP19A1 is a target for exemestane, an oral steroidal aromatase inhibitor approved by the FDA for the treatment of breast cancer in postmenopausal patients.^66^

The full-length model of CYP19A1 was generated by *e*Thread from the crystal structure of an N-terminal-truncated recombinant human CYP19A1 (PDB-ID: 4kq8, chain A, 100.0% sequence identity with a coverage of 89.9%).^67^ Subsequently, exemestane was placed in the steroid-binding pocket of CYP19A1 based on its global structure alignment with the X-ray structure of human placental CYP19A1 (PDB-ID: 3s79, chain A, TM-score of 0.89 and Cα-RMSD 0.55 Å) bound to androstenedione,^68^ another steroidal inhibitor with a TC to exemestane of 0.95. Although the experimental structure of CYP19A1 bound to exemestane is available (PDB-ID: 3s7s, chain A),^68^ it is not included in the template library used to model DrugBank complexes. By reason of removing the redundancy in the library at 80% protein sequence identity^33^ and a TC of 0.9 for the ligand chemical similarity,^34^ androstenedione-bound CYP19A1 was identified as a cluster centroid to represent the entire group of similar complexes, including the exemestane-CYP19A1 structure.

**Fig. 8:**
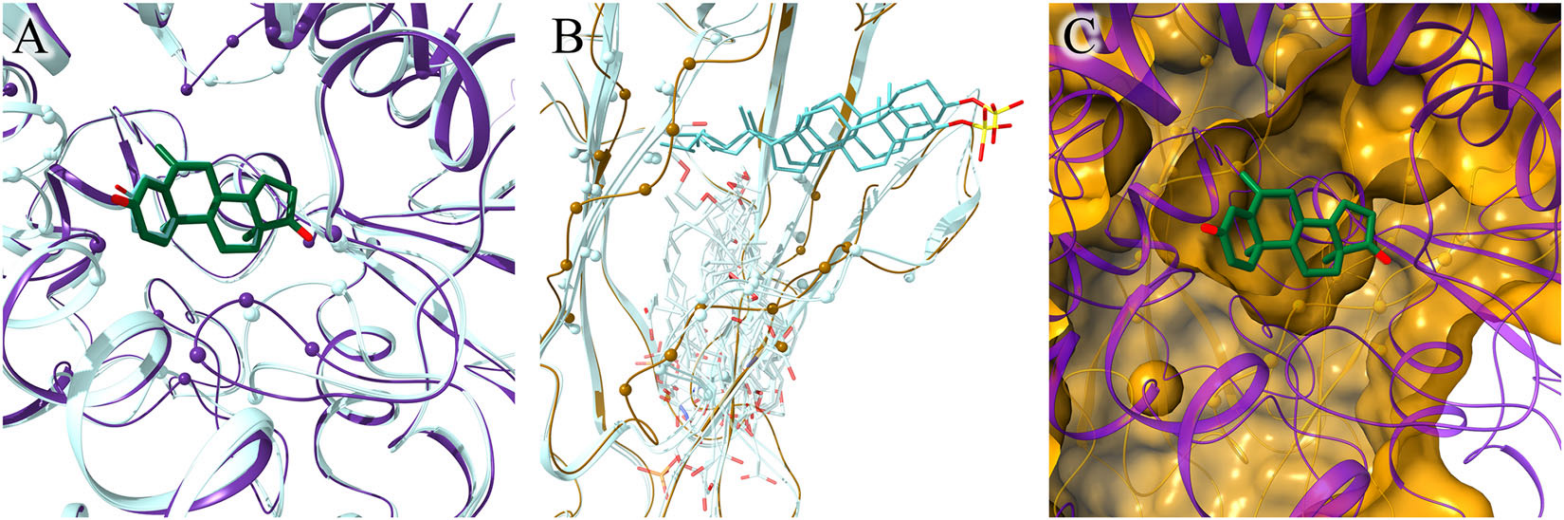
Repositioning of exemestane from cytochrome P450 aromatase (CYP19A1) to Niemann-Pick disease type C2 protein (NPC2). CYP19A1 and NPC2 proteins are colored in purple and gold, respectively, whereas ligands are colored by atom type (green/teal–carbon, red–oxygen, yellow–sulfur). **a** Global superposition of the modeled complex between CYP19A1 (purple ribbons) and exemestane (thick sticks), and two experimental structures of CYP19A1 (teal ribbons) bound to androstenedione and exemestane (thin sticks). Binding residues are shown as spheres. **b** Global superposition of the NPC2 model (gold ribbons) and two experimental structures of NPC2, human and bovine (teal ribbons), bound to cholesterol sulfate (thin sticks). Binding residues are shown as spheres. In addition, the steroid-binding pocket predicted by *e*FindSite is represented by a cluster of template-bound ligands (transparent sticks) extracted from the following template proteins superposed onto the NPC2 model (template-proteins are not shown): GM2A (PDB-IDs: 2ag2, 1tjj, 2agc), LY96 (PDB-IDs: 2e59, 2e56, 4g8a, 3fxi, 2z65, 3mu3, 3rg1, 5ijd, 3vq2, 3m7o), DERF2 (PDB-ID: 1xwv), and NPC2 (PDB-IDs: 5kwy,2hka, 3web). **c** Cross section of the internal cavity in the NPC2 structure exposing the repositioned exemestane (thick sticks). CYP19A1 (purple ribbons) and NPC2 (gold surface) are locally superposed according to the sequence order-independent pocket alignment by *e*MatchSite. Annotated binding residues in NPC2 are solid, whereas the remaining surface is transparent

We selected this case to demonstrate that a non-redundant library is adequate to build complex models fairly indistinguishable from experimental structures. The exemestane-CYP19A1 model constructed in this study is shown in Fig. 8a as thick sticks colored by atom type representing exemestane and purple ribbons representing CYP19A1. Two other structures are globally aligned onto the exemestane-CYP19A1 model, the androstenedione-CYP19A1 complex used as the template to position exemestane within the steroid-binding pocket and the experimentally determined exemestane-CYP19A1 complex; both structures are presented in Fig. 8a as thin sticks colored by atom type and teal ribbons. Indeed, the Cα-RMSD, as well as the RMSD calculated over binding residues between CYP19A1 model and experimental structure are below 1 Å. Further, RMSD calculated for exemestane upon the global structure superposition is as low as 0.06 Å demonstrating that the exemestane-CYP19A1 assembly is modeled with a very high accuracy. It is also noteworthy that *e*FindSite identified the binding site for exemestane with 96.2% confidence and the predicted binding residues, shown as purple spheres in Fig. 8a, yield an MCC of 0.71 against exemestane-binding residues in the CYP19A1 model.

The full-length model of lysosomal protein NPC2 was constructed based on the crystal structure of the human NPC2 (PDB-ID: 5kwy, chain C, 100.0% sequence identity with a coverage of 87.4%).^65^ Figure 8b shows the global superposition of the NPC2 model represented by gold ribbons and two experimental NPC2 structures represented by teal ribbons, human (the template, 5kwyC) and bovine (PDB-ID: 2hka, chain A, 79% sequence identity to human NPC2),^69^ both complexed with cholesterol sulfate. These superpositions yield a Cα-RMSD of 0.92 Å against human and 1.06 Å against bovine structures. NPC2 has an Ig-like β-sandwich fold comprising seven β-strands forming a hydrophobic pocket that was suggested to become wider in order to accommodate cholesterol-like molecules.^70^ This region was accurately identified by *e*FindSite with a high confidence of 95.2% as a highly hydrophobic binding site formed by 20 conserved residues. The prediction was made based on a non-redundant set of 21 holo-templates, including ganglioside GM2 activator (GM2A), lymphocyte antigen 96 (LY96), mite group 2 allergen Der f 2 (DERF2), and NPC2 itself. Selected template-bound ligands are shown in Fig. 8b as a cluster of transparent, teal molecules upon the global alignment of template proteins onto the NPC2 model. In addition, *e*FindSite estimated that the average ± standard deviation molecular weight (MW), octanol-water partition coefficient (logP), and polar surface area (PSA) for molecules binding to this region on the NPC2 surface are 383 Da ± 225, 4.76 ± 1.97, and 90.2 Å^2^ ± 77.9, respectively. The predicted physicochemical properties of putative binders of NPC2 are a good match for exemestane (and androstenedione), whose MW is 296 Da (286 Da), logP is 4.03 (4.09), and PSA is 34.1 Å^2^ (34.1 Å^2^).

Although the global similarity between CYP19A1 and NPC2 is low as assessed by a TM-score of 0.14 and 5.2% sequence identity, *e*MatchSite predicted that their binding sites are in fact similar with a high *e*MS-score of 0.86. Figure 8c shows exemestane repositioned from CYP19A1 to the cholesterol-binding pocket of NPC2 based on the sequence order-independent local alignment reported by *e*MatchSite. Exemestane fits into a deep, non-polar cavity in the NPC2 structure forming a number of hydrophobic interactions with Y55, V57, V73, V74, F85, P88, Y109, N111, L113, V126, W128, and W141. Encouragingly, an interaction energy of −409.5 calculated with DFIRE for the exemestane-NPC2 complex is lower than a value of −381.4 for exemestane-CYP19A1 indicating that this drug may form favorable interactions with NPC2. Notably, exemestane adopts a conformation distinct from that of cholesterol sulfate in the crystal structure of NPC2. The latter is larger (MW of 466 Da) and has two moieties attached to the steroid scaffold, an aliphatic branched-chain interacting with the inner part of the NPC2 pocket and a polar sulfate group protruding from the pocket toward the cholesterol-transfer tunnel between NPC2 and the N-terminal domain of NPC1.^65^ In contrast, smaller exemestane may bind deeper in the NPC2 structure to inhibit conformational changes required for transporting cholesterol to NPC1.

This conjecture is supported by several recent studies. For instance, U18666A, a cationic sterol similar to exemestane with a TC of 0.67, binds to NPC1, inhibiting cholesterol export.^71^ Further, FDA-approved ezetimibe was shown to target NPC1 decreasing the cholesterol level.^72^ Another study independently suggests repurposing thiabendazole, a potent inhibitor of cytochrome P450 1A2 (CYP1A2), to NPC1.^73^ Note that CYP1A2 and CYP19A1 are members of the cytochrome P450 family^74^ (Pfam-ID: PF00067) and have highly similar structures with a TM-score of 0.87. Finally, NPC2 was demonstrated to bind a range of cholesterol-related molecules, leading to an alteration of its function in lysosomal cholesterol transport.^75^ On that account, we hypothesize that exemestane binding to NPC2 disrupts the dynamics of its hydrophobic cavity. This effect could be exploited as a viable strategy to impede sterol movement to NPC1 preventing the accumulation of cholesterol in lysosomes in NPC disease.

## Conclusions

Rational repositioning of existing drugs is expected to play a major role in the development of treatments for orphan diseases. Comparing ligand-binding sites in protein structures is among the most promising computational techniques to inform drug repurposing efforts. In this study, we demonstrate that combining *e*MatchSite with structure-based virtual screening enhances the accuracy of the detection of similar binding pockets. This promising methodology was employed to match drug-binding pockets from DrugBank with those from Orphanet exposing a number of opportunities to combat orphan diseases with existing drugs.

## Materials and methods

### DrugBank and Orphanet datasets

The DrugBank dataset includes proteins binding FDA-approved drugs with a molecular weight of 150–550 Da selected from DrugBank,^32^ whereas the Orphanet dataset contains proteins associated with rare disorders obtained from Orphanet (http://www.orpha.net). Target structures composed of 50–999 amino acids in both datasets were modeled with *e*Thread, a template-based structure prediction algorithm.^33^ In the next step, drug-binding pockets were predicted by *e*FindSite^34^ in confidently modeled target DrugBank and Orphanet proteins whose estimated GDT-score is ≥0.4. Drug repositioning utilizes only those binding sites assigned a high and moderate confidence. Further, we devised a two-step alignment protocol to position drug compounds within the predicted binding pockets in the DrugBank proteins. First, holo-templates selected by *e*FindSite were structurally aligned onto the target protein with Fr-TM-align^35^ and then the drug molecule was superposed onto the most similar template-bound ligand according to the chemical alignment constructed by KCOMBU.^36^ The Orphanet dataset comprises 922 proteins, whereas the DrugBank dataset contains 2012 drug-protein complexes formed by 715 drugs and 348 proteins.

### Matching DrugBank and Orphanet pockets

All-against-all matching of drug-binding pockets in DrugBank and Orphanet proteins was conducted with *e*MatchSite.^15,16^ This algorithm constructs sequence order-independent alignments of pocket residues by solving the assignment problem with machine learning and the Kuhn-Munkres algorithm.^76,77^ Local alignments are then assigned a similarity score, called the *e*MS-score, which measures the overlap of various physicochemical features and evolutionary profiles. For significant matches identified with *e*MatchSite, drugs bound to the DrugBank target were transferred to a binding site in the Orphanet protein upon the superposition of the two pockets according to the local alignment. Subsequently, the constructed complexes of drugs repositioned to Orphanet proteins were rebuilt with Modeller^37^ in order to refine drug-target interactions eliminating steric clashes. The quality of final complex models is assessed by a knowledge-based statistical energy function for protein-ligand complexes with DFIRE^38^ and VS with Vina.^17^

### Huang dataset

The Huang dataset was originally compiled to evaluate the performance of geometry-based methods to predict binding pockets^21^ and then it was adopted to assess the accuracy of pocket comparison algorithms.^78^ From this dataset, we selected 107 proteins for which *e*FindSite correctly annotated binding sites within a distance of 8 Å from the geometric center of the bound ligand in the experimental complex structure. These target proteins bind the following ligands, adenosine, biotin, fructose-6-phosphate, α-L-fucose, β-D-galactose, guanine, α-D-mannose, O1-methyl-mannose, 4-phenyl-1H-imidazole, palmitic acid, retinol, and 2’-deoxyuridine 5’-monophosphate. The comprehensive information on the Huang dataset is given in Supplementary Table S1.

### Virtual screening

A target binding site is subjected to VS with AutoDock Vina^17^ against a non-redundant library of 1515 FDA-approved drugs compiled previously.^20^ MGL tools^79^ and Open Babel^80^ were used to add polar hydrogens and partial charges, as well as to convert target proteins and library compounds to the PDBQT format. For each docking ligand, the optimal search space centered on the binding site annotated with *e*FindSite was calculated from its radius of gyration.^81^ Molecular docking was carried out with AutoDock Vina 1.1.2 and the default set of parameters.

### Data availability

Data generated for the repositioning of DrugBank drugs to Orphanet proteins are available from the Open Science Framework at https://osf.io/qdjup/. The source codes of programs used in this study are available from GitHub, *e*Thread: https://github.com/michal-brylinski/ethread, *e*FindSite: https://github.com/michal-brylinski/efindsite, and *e*MatchSite: https://github.com/michal-brylinski/ematchsite.

## Electronic supplementary material

Supplementary Information

## Electronic supplementary material

**Supplementary Information** accompanies the paper on the *npj Systems Biology and Applications* website (10.1038/s41540-018-0050-7).
