# Supplementary material for: Large-scale computational drug repositioning to find treatments for rare diseases
Source: NPJ Syst Biol Appl. 2018 Mar 13;4:13. doi: 10.1038/s41540-018-0050-7 (PMC5847522; doi:10.1038/s41540-018-0050-7)
Supplement: Supplementary file 1 — Supplementary Information [file 41540_2018_50_MOESM1_ESM.pdf]

Supplementary Information

Large-scale computational drug repositioning to find treatments for rare diseases

by

Rajiv Gandhi Govindaraj[1], Misagh Naderi[1], Manali Singha[1], Jeffrey Lemoine[1,2], and Michal Brylinski[1,3*]

[1]Department of Biological Sciences, Louisiana State University, Baton Rouge, LA 70803, USA

[2]Division of Computer Science and Engineering, Louisiana State University, Baton Rouge, LA 70803, USA

[3]Center for Computation & Technology, Louisiana State University, Baton Rouge, LA 70803, USA

Table of content:

- **Text S1.** Measuring the chemical correlation with virtual screening (page 2).
- **Text S2.** Addressing the early recognition problem with BEDROC (page 2).
- **Text S3.** Evaluating the structure quality with RMSD, TM score, and GDT-score (page 3).
- **Text S4.** Ligand-binding site alignment with *e*MatchSite (page 3).
- **Text S5.** DFIRE statistical energy function for biomolecular complexes (page 4).
- **Text S6.** Ligand-binding site prediction with *e*FindSite (page 4).
- **Text S7.** Chemical alignment with KCOMBU and the Tanimoto coefficient (page 5).
- **Figure S1.** Interaction diagrams for models of ponatinib repositioned to Rab-23 (Page 6).
- **Figure S2.** Statistics for multiple models generated for drug-Orphanet complexes (Page 7).
- **Table S1.** The Huang dataset of bound and unbound proteins (page 8).
- **References** (page 13).

Text S1. Measuring the chemical correlation with virtual screening.

The chemical correlation was developed to indirectly measure the similarity between binding sites with virtual screening. [1,2] It can be calculated for compound ranks assigned by either ligand- or structure-based virtual screening. In the present study, structure-based virtual screening is conducted with AutoDock Vina [3] for target pocket in the Huang dataset [4] against a non-redundant library of 1,515 FDA-approved drugs obtained from the DrugBank database. [5] Docking poses generated by Vina are ranked according to the predicted binding energy. Subsequently, non-parametric Spearman's ρ correlation coefficient [6] is computed for compound ranks assigned to a pair of pockets. Spearman's ρ measures the degree of monotonic relationship ranging from +1 to -1, where +1 is a perfect correlation, 0 is the lack of any correlation, and -1 is an anti-correlation. A high Spearman's ρ indicates that a pair of pockets not only exhibit high binding affinity toward similar compounds but also do not bind similar ligands.

Text S2. Addressing the early recognition problem with BEDROC.

The Boltzmann-enhanced discrimination of the receiver operating characteristic (BEDROC) [7] is a generalization of the area under the ROC curve (AUC) addressing the early recognition problem. While the AUC metric is useful to assess the performance of a binary classifier, it fails to discriminate curves with the same AUCs but differing degrees of the early recall. For two ROC curves varying in shape, many applications prefer the curve with a higher proportion of its AUC at a low false positive rate. Classifiers requiring early recognition capabilities include, for instance, virtual screening and the detection of off-targets, where a large number of initial molecules must be reduced to a testable number of promising candidates. Similar to AUC, BEDROC ranges from 0 to 1 and can be interpreted as the probability of a ranked positive to be positioned higher in the ordered list than by a random chance. However, in contrast to the uniform distribution in AUC, BEDROC is based on the exponential distribution with the adjustable exponential factor defining the desired degree of "early recognition". In our study, we use the recommended value of 20, which means that 80% of the maximum contribution to the BEDROC score comes from the first 8% of the ranked list.

Text S3. Evaluating the structure quality with RMSD, TM-score, and GDT-score.

The root-mean-square deviation (RMSD) measures the similarity between superposed three-dimensional protein structures based on Cartesian distances. [8] It can be calculated for Cα atoms or all atoms over the entire length of a protein, as well as for specific regions, such as transmembrane helices, loops, binding pockets, etc. The unit of the RMSD is Angstrom [Å] and high values correspond to low similarities between two structures. Nonetheless, the global RMSD was shown to be the least representative of the degree of structural similarity because it is dominated by the largest error, [9] for instance, different conformation of a single loop can inflate the RMSD between two otherwise identical proteins. Furthermore, the RMSD is strongly length-dependent complicating the comparison of proteins of different length.

A number of other measures have been developed to provide a statistically meaningful assessment of similarity between biomolecules. An example is the Template Modeling (TM)-score quantifying the topological similarity between a pair of protein structures based on the coordinates of Cα atoms. [10] TM-score ranges from 0 to 1 with higher values indicating a higher similarity between protein structures, and the value of 1 is a perfect match between two structures. Scores below 0.17 correspond to randomly chosen unrelated protein structures, whereas scores above 0.5 indicate that two protein structures have the same fold [11] according to the Structural Classification of Proteins (SCOP) [12] and the CATH Protein Structure Classification database. [13] Another metric is the Global Distance Test (GDT)-score reporting the number of Cα atom pairs within distance thresholds of 1, 2, 4, and 8 Å after the superimposition of the query and reference structures. [14] However, these distance cutoffs are subjective and may require target-specific adjustments. [15] Further, the magnitude of the GDT-score for random structure pairs has a similar to the RMSD power-law dependence with the protein length. [10] GDT-score ranges from 0 to 1 with higher values indicating a higher similarity between protein structures.

Text S4. Ligand-binding site alignment with *e*MatchSite.

*e*MatchSite is a sequence-order independent algorithm to compare ligand-binding sites. [1,16] It assigns a set of residue-level scores extracted from weakly homologous template proteins

complexed with small molecules covering various properties of binding ligands and residues. In addition, the evolutionary information is included as sequence and secondary structure profiles, and entropy. An important feature of *e*MatchSite is its capability to predict pairwise $C\alpha$-$C\alpha$ distances between binding residues upon the optimal alignment of two pockets by machine learning. Based on these distances, it constructs local alignments of pocket residues by solving the assignment problem with the Kuhn-Munkres algorithm. [17,18] Binding site alignments are subsequently assigned a similarity score, called the *e*MS-score, which measures the overlap of various physicochemical and evolutionary features. *e*MS-score ranges from 0 for completely dissimilar pockets to 1 for identical pockets, with an optimized threshold of 0.56 accurately distinguishing between pockets binding similar and dissimilar molecules.

Text S5. DFIRE statistical energy function for biomolecular complexes.

The goal of the modeling of ligand-protein interactions is to identify biologically relevant, near-native complexes. An important component of the modeling procedure is the prediction of the energy of association between small molecules and their macromolecular targets. This task can be accomplished by physics-based, knowledge-based, or empirical scoring functions. Distance-scaled Finite Ideal-gas REference (DFIRE) is a knowledge-based statistical potential to predict binding affinities for ligand-protein, protein-protein, and DNA-protein complexes. Binding affinities estimated by DFIRE are highly correlated with those experimentally determined with a Pearson correlation coefficient R of 0.63, outperforming 12 other scoring functions. This energy function also offers highly accurate predictions of binding affinities for protein-protein (R = 0.73) and DNA-protein (R = 0.83) complexes. Because of the high accuracy of DFIRE, we employ this scoring function to evaluate binding energies of drugs repositioned to off-target proteins with *e*MatchSite.

Text S6. Ligand-binding site prediction with *e*FindSite.

*e*FindSite is a structure/evolution-based ligand-binding site prediction approach employing meta threading to identify a set of evolutionarily related templates complexed with ligands. [19,20] These templates are first structurally aligned onto the target with Fr-TM-align [21] followed by the

clustering of the centers of mass of bound ligands to identify putative binding sites in the target structure. *e*FindSite offers a machine learning-based confidence estimation system not only to rank the predicted sites, but also to reliably evaluate the corresponding ranking confidence. This algorithm uses a vector of various features, including the fraction of templates that share a particular site, the cluster multiplicity, the average TM-score of templates to the target, the number and the average confidence of predicted binding residues, and a protein-ligand binding index calculated over predicted binding residues. The assigned confidence estimates the likelihood that the site center is predicted within a distance of 8 Å from the geometrical center of a natively bound ligand.

Text S7. Chemical alignment with KCOMBU and the Tanimoto coefficient.

Comparing the chemical structures of organic molecules has a number of applications in cheminformatics. Techniques employing the graph theory find equivalent atom and bonds in molecules by solving the maximum common substructure (MCS) and/or maximum clique problems. An example of such algorithm is the K(ch)emical structure COMparison using the BUild-up algorithm (KCOMBU). [22] This method is capable of finding connected and disconnected MCSs in molecules represented by graphs. In addition to the chemical alignment between two molecules, KCOMBU reports their similarity in terms of the Tanimoto coefficient (TC). [23] Widely used TC is arguably the most reliable similarity measure for low-molecular weight organic molecules [24]. Briefly, the TC compares the extent of commonality or similarity between two sets by defining the ratio of common elements to the non-common elements. TC ranges from 0 for a pair of completely dissimilar compounds to 1 indicating identical molecules. For molecule pairs with the TC greater than 0.4, KCOMBU was demonstrated to correctly match the majority of atoms when compared to their exact 3D superpositions. Therefore, a minimum TC value of 0.4 in KCOMBU should be employed keeping in mind that the atom matching accuracy significantly improves for chemical alignments assigned higher TC values.

Figure S1. Interaction diagrams generated by PoseView [25] for multiple models of a drug-target complex constructed based on multiple pocket alignments. Ponatinib is repositioned to Ras-related protein Rab-23 based on its local alignment with (**A**) Lck/Yes-related novel protein tyrosine kinase, (**B**) lymphocyte cell-specific protein-tyrosine kinase, and (**C**) proto-oncogene tyrosine-protein kinase Src. Hydrogen bonds are depicted by black dashed lines, aromatic interactions are indicated by green dashed lines connecting green solid dots at the aromatic ring centers, and hydrophobic interactions are illustrated as smooth, green contour lines.

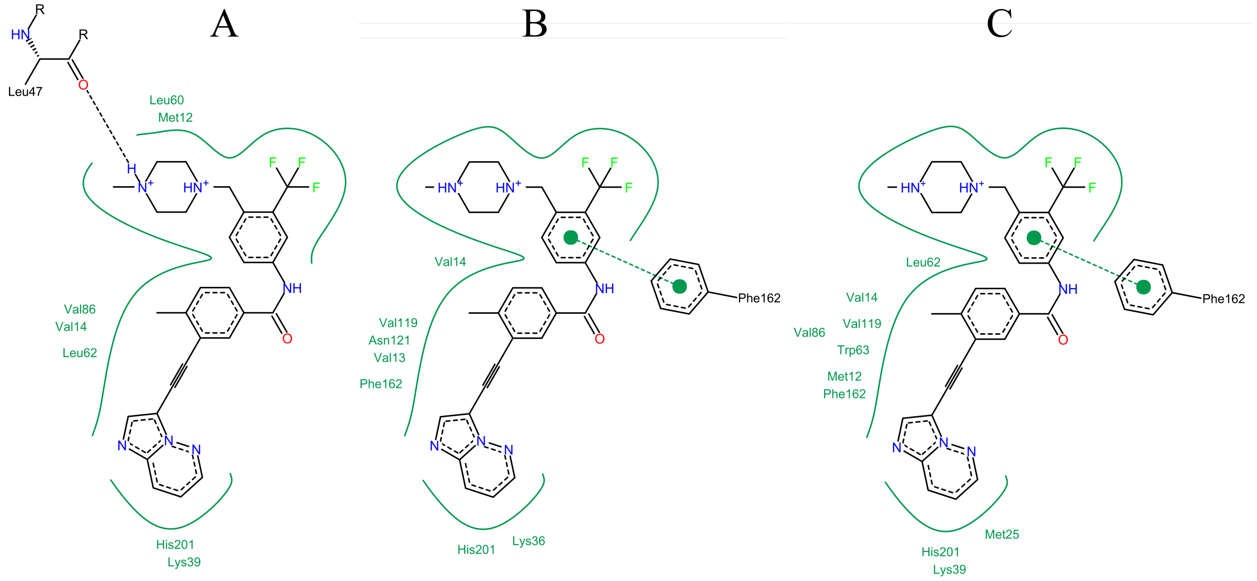

Figure S2. Histogram of the number of structure models generated for a subset of 4,878 drug-Orphanet complexes. Multiple structure models of the same complex are constructed using pocket alignments between the Orphanet target and different DrugBank proteins. **Inset:** Histogram of RMSD values calculated for different models of the same drug-target complex. RMSD is the root-mean-square deviation computed over ligand heavy atoms.

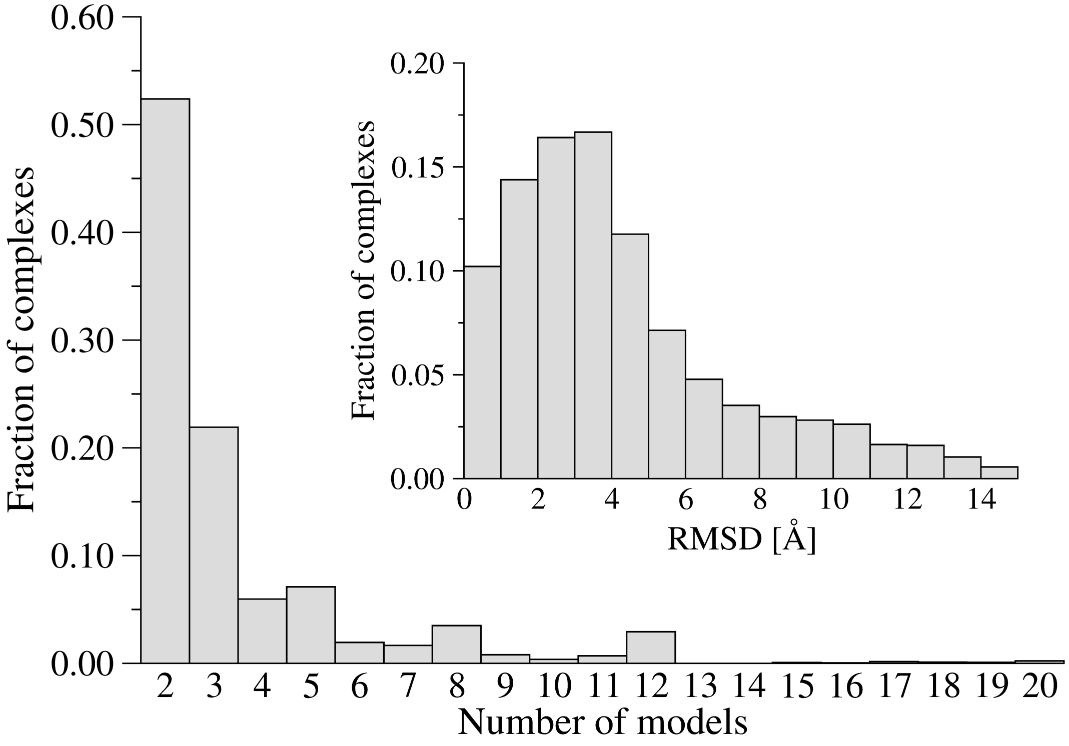

Table S1. The Huang dataset of bound and unbound proteins.

Ligand	Structure	PDB-ID	Chain	Protein
Adenosine (ADN) 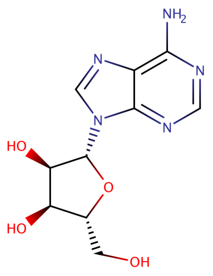	Bound	1fmo	E	cAMP-dependent protein kinase
		1pg2	A	Methionyl-tRNA synthetase (MetRS)
		1vhw	A	Dimethyladenosine transferase
		2eva	A	Transforming growth factor β-activated kinase 1 (TAK1) kinase adaptor
		2fqy	A	Membrane lipoprotein tmpc
		2pgf	A	Adenosine deaminase
		3ce6	A	Adenosylhomocysteinase
		3fuu	A	Dimethyladenosine transferase
	Unbound	3fut	A	Dimethyladenosine transferase
Biotin (BTN) 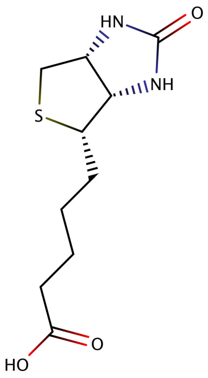	Bound	1bdo	A	*Acetyl-CoA carboxylase*
		1hxd	A	Bira bifunctional protein
		1stp	A	Streptavidin complex with biotin
		2b8g	A	Biotin/lipoyl attachment protein
		2f01	A	Streptavidin
		2jgs	A	Circular permutant of avidin
		3ew2	A	Rhizavidin
	Unbound	1swb	A	Streptavidin
Fructose-6-phosphate (F6P) 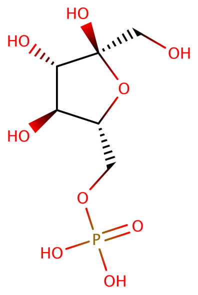	Bound	3fut	A	Central glycolytic gene regulator
		3bxh	A	Mannose-6-phosphate isomerase
		3h1y	A	N-acetylglucosamine-6-phosphate deacetylase
		3iv8	A	Glucose-6-phosphate isomerase
		2cxs	A	Phosphoenzyme intermediate of fru-2,6-bisphosphatase
		1tip	A	Fructose-1,6-bisphosphatase
		1nuy	A	Fructose 1,6-bisphosphatase/inositol monophosphatase
		1lby	A	Central glycolytic gene regulator
	Unbound	2fbp	A	Fructose 1,6-bisphosphatase

Fucose (FUC) 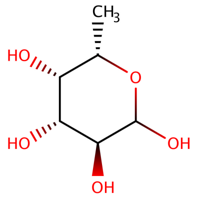	Bound	1k12	A	Lectin
		1uzv	A	Pseudomonas aeruginosa lectin ii
		2j1t	A	Fucolectin-related protein
		3cqo	A	Fbp32
		3kmb	1	Phosphoenzyme intermediate of fru-2,6-bisphosphatase
	Unbound	1kmb	1	Mannose-binding protein-a
Galactose (GAL) 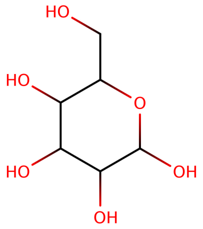	Bound	1axz	A	Lectin
		1gca	A	Glucose/galactose-binding protein
		1jz7	A	β-galactosidase
		1kwk	A	β-galactosidase
		1muq	A	Galactose-specific lectin
		1oko	1	Pa-I galactophilic lectin
		1r47	J	α-galactosidase A
		1rdk	A	Mannose-binding protein-c
		1rvt	A	Hemagglutinin
		1tlg	A	β-galactosidase
		1xc6	A	Glucose-binding protein
		2b3f	A	Polyandrocarpa lectin
		2e9m	A	Cytosolic β-glucosidase
		2gal	A	Galectin-7
		2j1a	A	Hyaluronidase
		2j5z	A	Ficolin-3
		2rjo	A	Twin-arginine translocation pathway signal protein
		2v72	B	Exo-α-sialidase
		2vjj	B	Tailspike protein
		2vno	A	Cpe0329
		2zgn	A	Anti-tumor lectin
		3a23	A	Putative secreted α-galactosidase
		3c69	A	Uncharacterized protein ygjk
		5abp	A	L-arabinose-binding protein
	Unbound	1gcg	A	Galactose/glucose-binding protein

Guanine (GUN) 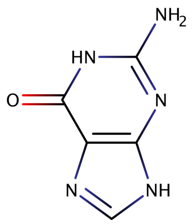	Bound	1a95	C	Xanthine-guanine phosphoribosyltransferase
		1d6a	A	Pokeweed antiviral protein
		1it7	A	Archaeosine trna-guanine transglycosylase
		1wet	A	Protein (purine repressor)
		1xe7	A	Hypothetical 22.5 kda protein in tub1-cpr3 intergenic region
		2i9u	A	Cytosine/guanine deaminase related protein
		2o74	A	Ohcu decarboxylase
		2ood	A	Blr3880 protein
		2puc	A	Protein (purine repressor)
		2puf	A	Protein (purine repressor)
		3bp1	B	NADPH-dependent 7-cyano-7-deazaguanine reductase
	Unbound	1ula	A	Purine nucleoside phosphorylase
Mannose (MAN) 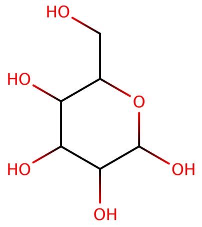	Bound	1g12	A	Peptidyl-lys metalloendopeptidase
		1js8	A	Hemocyanin
		1kza	1	Mannose-binding protein c
		1qmo	A	Mannose binding lectin, fril
		1rin	A	Pea lectin
		1xxr	B	Mannose-binding lectin
		2duq	A	Vesicular integral-membrane protein vip36
	Unbound	2duo	A	Vesicular integral-membrane protein vip36
O1-methyl mannose (MMA) 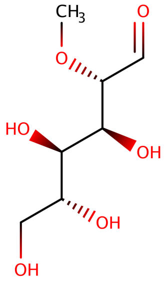	Bound	1kiu	B	Chaperone protein fimc
		1kwu	A	Mannose-binding protein a
		1lob	A	Legume isolectin i (α chain)
		1msa	A	Agglutinin
		1mvq	A	Lectin, isoform 1
		1rdl	1	Mannose-binding protein-c
		2bv4	A	Lectin cv-iil
		3g81	A	Pulmonary surfactant-associated protein d
	Unbound	2ctv	A	Concanavalin A

2-Phenylimidazol (PIM) 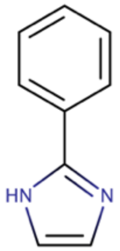	Bound	1e9x	A	Cytochrome p450 51-like rv0764c
		1f4t	A	Cytochrome P450 119
		1phd	A	Cytochrome p450-cam
		1s1f	A	Putative cytochrome p450
		2d0t	A	Indoleamine 2,3-dioxygenase
	Unbound	1phc	A	Cytochrome p450-cam
Palmitic Acid (PLM) 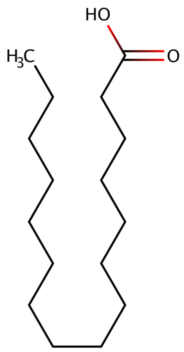	Bound	1eh5	A	Palmitoyl protein thioesterase 1
		1gxa	A	β-lactoglobulin
		1hxs	1	Genome polyprotein, coat protein vp1
		1lv2	A	Hepatocyte nuclear factor 4-γ
		1sz7	A	Trafficking protein particle complex subunit 3
		2dt8	A	Degv family protein
		2e9l	A	Cytosolic β-glucosidase
		2go3	A	Udp-3-o-[3-hydroxymyristoyl] n-acetylglucosamine deacetylase
		2uwh	A	Bifunctional p-450\: NADPH-p450 reductase
		3bfh	A	Pheromone-binding protein asp1
		3cue	E	Transport protein particle 23 kda subunit
		3egl	A	DegV family protein
		3epy	A	Acyl-coA-binding domain-containing protein 7
	Unbound	1ifb	A	Intestinal fatty acid binding protein
Retinol (RTL) 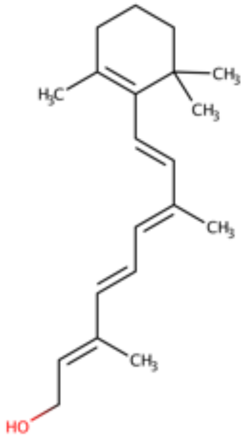	Bound	1fmj	A	Retinol dehydratase
		1gx8	A	β-lactoglobulin
		1kt6	A	Plasma retinol-binding protein
		2rct	A	Retinol-binding protein ii, cellular
	Unbound	1brq	A	Retinol binding protein

2’-deoxyuridine-5-monophosphate (UMP) 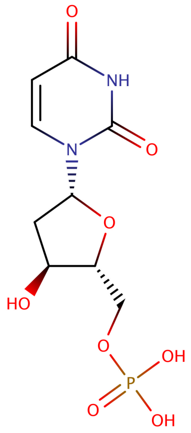	Bound	1f7n	A	Pol polyprotein
		1seh	A	Deoxyuridine 5'-triphosphate nucleotidohydrolase
		2bsy	A	Deoxyuridine 5'-triphosphate nucleotidohydrolase
		2g8o	A	Thymidylate synthase
		2jar	A	5'(3')-deoxyribonucleotidase
		2qch	A	Uridine 5'-monophosphate synthase (UMP synthase)
		3dl5	A	Dihydrofolate reductase, DHFR
	Unbound	3tms	A	Thymidylate synthase

References

1 Brylinski, M. eMatchSite: sequence order-independent structure alignments of ligand binding pockets in protein models. *PLoS Comput Biol* **10**, e1003829, doi:10.1371/journal.pcbi.1003829 (2014).

2 Brylinski, M. & Skolnick, J. Cross-reactivity virtual profiling of the human kinome by X-react(KIN): a chemical systems biology approach. *Mol Pharm* **7**, 2324-2333, doi:10.1021/mp1002976 (2010).

3 Trott, O. & Olson, A. J. AutoDock Vina: improving the speed and accuracy of docking with a new scoring function, efficient optimization, and multithreading. *J Comput Chem* **31**, 455-461, doi:10.1002/jcc.21334 (2010).

4 Huang, B. & Schroeder, M. LIGSITEcsc: predicting ligand binding sites using the Connolly surface and degree of conservation. *BMC Struct Biol* **6**, 19, doi:10.1186/1472-6807-6-19 (2006).

5 Wishart, D. S. *et al.* DrugBank: a comprehensive resource for in silico drug discovery and exploration. *Nucleic Acids Res* **34**, D668-672, doi:10.1093/nar/gkj067 (2006).

6 Corder, G. W. & Foreman, D. I. *Nonparametric statistics for non-statisticians: A step-by-step approach*. (John Wiley & Sons, Inc., 2009).

7 Truchon, J. F. & Bayly, C. I. Evaluating virtual screening methods: good and bad metrics for the "early recognition" problem. *J Chem Inf Model* **47**, 488-508, doi:10.1021/ci600426e (2007).

8 Kabsch, W. A solution for the best rotation to relate two sets of vectors. *Acta Crystallogr A* **32**, 922-923, doi:doi:10.1107/S0567739476001873 (1976).

9 Kufareva, I. & Abagyan, R. Methods of protein structure comparison. *Methods Mol Biol* **857**, 231-257, doi:10.1007/978-1-61779-588-6_10 (2012).

10 Zhang, Y. & Skolnick, J. Scoring function for automated assessment of protein structure template quality. *Proteins* **57**, 702-710, doi:10.1002/prot.20264 (2004).

11 Xu, J. & Zhang, Y. How significant is a protein structure similarity with TM-score = 0.5? *Bioinformatics* **26**, 889-895, doi:10.1093/bioinformatics/btq066 (2010).

12 Lo Conte, L. *et al.* SCOP: a structural classification of proteins database. *Nucleic Acids Res* **28**, 257-259 (2000).

13 Orengo, C. A. *et al.* CATH - a hierarchic classification of protein domain structures. *Structure* **5**, 1093-1108 (1997).

14 Zemla, A., Venclovas, C., Moult, J. & Fidelis, K. Processing and analysis of CASP3 protein structure predictions. *Proteins* **Suppl 3**, 22-29 (1999).

15 Kopp, J., Bordoli, L., Battey, J. N., Kiefer, F. & Schwede, T. Assessment of CASP7 predictions for template-based modeling targets. *Proteins* **69 Suppl 8**, 38-56, doi:10.1002/prot.21753 (2007).

16 Brylinski, M. Local alignment of ligand binding sites in proteins for polypharmacology and drug repositioning. *Methods Mol Biol* **1611**, 109-122, doi:10.1007/978-1-4939-7015-5_9 (2017).

17 Kuhn, H. W. The Hungarian method for the assignment problem. *Naval Research Logistics Quarterly* **2**, 83-97 (1955).

18 Munkres, J. Algorithms for the assignment and transportation problems. *Journal of the Society for Industrial and Applied Mathematics* **5**, 32-38 (1957).
19 Brylinski, M. & Feinstein, W. P. eFindSite: improved prediction of ligand binding sites in protein models using meta-threading, machine learning and auxiliary ligands. *J Comput Aided Mol Des* **27**, 551-567, doi:10.1007/s10822-013-9663-5 (2013).
20 Feinstein, W. P. & Brylinski, M. eFindSite: Enhanced fingerprint-based virtual screening against predicted ligand binding sites in protein models. *Mol Inform* **33**, 135-150, doi:10.1002/minf.201300143 (2014).
21 Pandit, S. B. & Skolnick, J. Fr-TM-align: a new protein structural alignment method based on fragment alignments and the TM-score. *BMC Bioinformatics* **9**, 531, doi:10.1186/1471-2105-9-531 (2008).
22 Kawabata, T. Build-up algorithm for atomic correspondence between chemical structures. *J Chem Inf Model* **51**, 1775-1787, doi:10.1021/ci2001023 (2011).
23 Tanimoto, T. T. An elementary mathematical theory of classification and prediction., (IBM Internal Report, 1958).
24 Bajusz, D., Racz, A. & Heberger, K. Why is Tanimoto index an appropriate choice for fingerprint-based similarity calculations? *J Cheminform* **7**, 20, doi:10.1186/s13321-015-0069-3 (2015).
25 Stierand, K. & Rarey, M. Drawing the PDB: Protein-ligand complexes in two dimensions. *ACS Med Chem Lett* **1**, 540-545, doi:10.1021/ml100164p (2010).
